# Predictive models to estimate utility from clinical questionnaires in schizophrenia: findings from EuroSC

**DOI:** 10.1007/s11136-015-1120-6

**Published:** 2015-09-18

**Authors:** Carole Siani, Christian de Peretti, Aurélie Millier, Laurent Boyer, Mondher Toumi

**Affiliations:** Research Laboratory in Knowledge Engineering (ERIC, EA3083), Institute of Pharmaceutical and Biological Sciences (ISPB), University Claude Bernard Lyon 1, 11 Rue Guillaume Paradin, 69372 Lyon Cedex 08, France; Laboratory of Actuarial and Financial Sciences (SAF, EA2429), Institute of Financial and Insurance Sciences (ISFA School), University Claude Bernard Lyon 1, 50 Avenue Tony Garnier, 69366 Lyon Cedex 7, France; Creativ-Ceutical, 215 Rue du Faubourg Saint Honoré, 75008 Paris, France; Aix-Marseille University, 27 bd Jean Moulin, 13385 Marseille Cedex 05, France; Laboratory of Public Health (EA 3279), Faculty of Medicine, Aix-Marseille University, 27 bd Jean Moulin, 13385 Marseille Cedex 05, France

**Keywords:** Schizophrenia, Quality of life, Positive and Negative Syndrome Scale, Mapping

## Abstract

**Objective:**

The clinical symptoms of schizophrenia are associated with serious social, quality of life and functioning alterations. Typically, data on health utilities are not available in clinical studies in schizophrenia. This makes the economic evaluation of schizophrenia treatments challenging. The purpose of this article was to provide a mapping function to predict unobserved utility values in patients with schizophrenia from the available clinical and socio-demographic information.

**Methods:**

The analysis was performed using data from EuroSC, a 2-year, multi-centre, cohort study conducted in France (*N* = 288), Germany (*N* = 618), and the UK (*N* = 302), totalling 1208 patients. Utility was calculated based on the EQ-5D questionnaire. The relationships between the utility values and the patients’ socio-demographic and clinical characteristics (Positive and Negative Syndrome Scale—PANSS, Calgary Depression Scale for Schizophrenia—CDSS, Global Assessment of Functioning—GAF, extra-pyramidal symptoms measured by Barnes Akathisia Scale—BAS, age, sex, country, antipsychotic type) were modelled using a random and a fixed individual effects panel linear model.

**Results:**

The analysis demonstrated the prediction ability of the used parameters for estimating utility measures in patients with schizophrenia. Although there are small variations between countries, the same variables appear to be the key predictors. From a clinical perspective, age, gender, psychopathology, and depression were the most important predictors associated with the EQ-5D.

**Conclusion:**

This paper proposed a reliable, robust and easy-to-apply mapping method to estimate EQ-5D utilities based on demographic and clinical measures in schizophrenia.

**Electronic supplementary material:**

The online version of this article (doi:10.1007/s11136-015-1120-6) contains supplementary material, which is available to authorized users.

## Introduction

Economic evaluation allows comparing several alternative therapies in terms of benefit brought by a new treatment and associated costs. The comparison is often conducted by the calculation of incremental cost-effectiveness ratio (ICER). Cost-utility analysis is a common type of economic evaluation in which product effectiveness is expressed in quality-adjusted life years (QALYs). QALYs are determined by multiplying life years gained using the therapy by utilities, corresponding to patient’s quality of life (QoL) during this period. Utility of a certain health state accounts for different aspects of QoL and corresponds with the desire or preference that individuals exhibit for this state. Unlike psychometric measures that consider only the degree of abnormality of an impairment of health, utility preference-based measures allow estimating the significance of the impairments and so can be used in economic evaluation to assess the value of interventions from the perspective of the patient. A number of instruments have been developed to measure preference-based utilities directly, or through generic or disease-specific preference-based questionnaires. However, in the context of developing cost-utility models, utility measurements are rarely available and are sometimes predicted using mapping extrapolation from a clinical questionnaire and other parameters [10].

Schizophrenia is chronic mental disorder which significantly impacts patient’s QoL [11, 12, 34]. It affects approximately 1 % of the general population, and onset usually occurs before the age of 25 years [28]. Schizophrenia symptoms are generally devised in positive symptoms (psychotic behaviours not seen in healthy people, i.e. hallucination, delusions, etc.), negative symptoms (decrease or loss of normal functions, i.e. apathy, lack of emotions), and cognitive symptoms. The key component of schizophrenia treatment is antipsychotic medications which are mainly used to manage positive symptoms. There exist two generations of antipsychotic drugs called typical (first generation) and atypical (second generation) antipsychotics.

There is a large range of symptoms and treatment-related adverse events, and these are associated with serious functioning, social, and QoL alterations in patients with schizophrenia. This makes the utility assessment challenging. In 2010, Mavranezouli reported that seven utility or cost-utility analyses were performed for schizophrenia treatment [29]. Out of these analyses, none of the studies used utility values for schizophrenia which had been generated by using EQ-5D, otherwise widely used in cost-utility analyses and a preferred instrument of the National Institute for Health and Clinical Excellence NICE [32]. Therefore, developing a predictive equation to estimate utility measures based on clinical, functioning, and QoL variables would support and simplify health economics assessment of therapies designated to treat patients suffering from schizophrenia.

Several studies have investigated the independent predictors of QoL in people with schizophrenia. These studies report that clinical factors, such as positive and negative symptoms, depression, and extra-pyramidal symptoms, are associated with low QoL [2, 14, 22, 27, 33, 36, 38, 39, 43].

The objective of this article is to generate a predictive equation for EQ-5D utility in schizophrenia which would allow its extrapolation from other instruments and clinical parameters, using data from the European Schizophrenia Cohort (EuroSC). Two predictive models were developed by using two sets of variables in order to take into account differences in the availability of clinical data to practitioners. Additionally, a predictive model for SF-6D utilities was developed to study mapping mechanisms and to determine whether mapping the same set of predictors to the utilities collected, using the two different instruments, conforms to a different algorithm.

## Data and methods

### Design and sample

The EuroSC is a European cohort conducted in France, Germany, and the UK with a prospective follow-up from 1998 to 2001. A total of 1208 participants were interviewed at 6-month intervals for a total of 2 years in France (*N* = 288), Germany (*N* = 618), and the UK (*N* = 302). This study was sponsored by H. Lundbeck A/S.

The first objective of the study was to identify and describe the types of treatments and methods of care for patients with schizophrenia and to correlate these treatments with clinical outcomes, states of health, and QoL. In each country, catchment areas were chosen based on socio-demographic factors and on the styles of service delivery. Nine European centres were considered: two in Britain, four in Germany, and three in France. The participants were selected to provide a representative sample of the patients treated in secondary psychiatric services in each catchment area.

Random sampling from these patients was used to generate a representative sample. This project was conducted in accordance with the Declaration of Helsinki and French Good Clinical Practices [5, 42]. A description of the rationales and methods of the study is presented in Bebbington et al. [7].

### Data collection and instruments

Collected data included utility measures, socio-demographic characteristics, and clinical and treatment information. Patients’ health states were assessed using Positive and Negative Syndrome Scale (PANSS) [9, 24], Calgary Depression Scale for Schizophrenia (CDSS) [1], and global assessment of functioning (GAF) [17], and extra-pyramidal symptoms were measured by Barnes Akathisia Scale (BAS) [6].

The utility measures were computed from the multi-attribute EuroQol EQ-5D questionnaire [13, 23, 40], using the British scoring formula. EQ-5D measures five dimensions: mobility, self-care, usual activities, pain/discomfort, and anxiety/depression. Each dimension has three categories of severity: indicating no problem, some problem, and extreme problems. Patients are asked to choose the severity level for each dimension that corresponds most to his health state according to his perception. In such a manner each health state is coded by a five-digit number, which can be derived into utility [15]. EQ-5D covers a total of 243 health states.

Together with EQ-5D, health-related QoL was assessed using SF-6D questionnaire [41]. The SF-6D is a revision of the SF-36 questionnaire, the most widely used generic instrument to measure general health in clinical studies, as the latter cannot be used for economic evaluation in its original form. The SF-6D allows for the economic evaluation and covers six domains including physical functioning, role limitation, social functioning, pain, mental health, and vitality. Any patient who completes the SF-36 can be classified according to the SF-6D derived value.

Available socio-demographic and treatment information included the country of origin (country), age of the patient at the date of the visit (age), gender (sex being 1 for males and 0 for females), and antipsychotic type (ATYP)—typical, atypical, or mixed (containing at least one typical antipsychotic and one atypical antipsychotic).

Symptoms severity was measured using PANSS, a comprehensive tool that includes 30 items and requires an individual interview with each patient (30–40 min). The items are assessed based on the patient’s perceptions of their experiences in the previous week. The interview covers the following domains: positive sub-score (PANSS_POS), negative sub-score (PANSS_NEG), and general psychopathology sub-score (PANSS_PSY). The PANSS_POS and PANSS_NEG contain seven and the PANSS_PSY contains 16 items all ranging from 1 (minimal problem) to 7 (extreme problem).

Depressive symptoms were assessed using CDSS, containing nine items with four possible severity levels (range from 1 to 27). Moreover, patient states were examined employing GAF scale, commonly used to assess psychological, social, and occupational functioning in mental health illness (range 1–100). Extra-pyramidal symptoms were assessed using BAS—a specific scale developed to determine the severity of drug-induced Akathisia. BAS includes objective and subjective items as well as global clinical assessment of the Akathisia score (range 1–100).

### Predictive models

Two predictive models were developed using two sets of independent variables. Both models are summarized in Eq. 1 and Eq. 2. Negative EQ-5D values were allowed to account for very severe health states; consequently, EQ-5D variable was ranged from minus infinite to one. The EQ-5D variable was then considered by approximation as a continuous variable. Since variables are observed by individuals and by time, panel models were considered as appropriate. In addition, fixed and random effects by patient were introduced in the statistical models to account for patients’ heterogeneity.

1 $$ \begin{aligned} {\text{EQ}} - 5Q_{it} & = \alpha + \beta_{1} \cdot {\text{PANSS}}\_{\text{POS}}_{it} + \beta_{2} \cdot {\text{PANSS}}\_{\text{NEG}}_{it} + \beta_{3} \cdot {\text{PANSS}}\_{\text{PSY}}_{it} + \beta_{4,1} \cdot {\text{Age}}_{it} \\ & \quad + \beta_{4,2} \cdot {\text{Age}}_{it}^{2} + \beta_{5} \cdot {\text{Sex}}_{i} + \beta_{6} \cdot {\text{FR}}_{i} + \beta_{7} \cdot {\text{GE}}_{i} + u_{i} + e_{it} , \\ \end{aligned} $$

2 $$ \begin{aligned} {\text{EQ}} - 5Q_{it} & = \alpha + \beta_{1} \cdot {\text{PANSS}}\_{\text{POS}}_{it} + \beta_{2} \cdot {\text{PANSS}}\_{\text{NEG}}_{it} + \beta_{3} \cdot {\text{PANSS}}\_{\text{PSY}}_{it} + \beta_{4,1} \cdot {\text{Age}}_{it} \\ & \quad + \beta_{4,2} \cdot {\text{Age}}_{it}^{2} + \beta_{5} \cdot {\text{Sex}}_{i} + \beta_{6} \cdot {\text{FR}}_{i} + \beta_{7} \cdot {\text{GE}}_{i} + \beta_{8} \cdot {\text{AP}}1_{it} + \beta_{9} \cdot {\text{AP}}2_{it} \\ & \quad + \beta_{10} \cdot {\text{CDSS}}_{it} + \beta_{11} \cdot {\text{GAF}}_{it} + \beta_{12} \cdot {\text{BAS}}_{it} + u_{i} + e_{it} , \\ \end{aligned} $$

$u_{i} \sim i.i.d.N\left( {0,\sigma_{i}^{2} } \right)$ for the random individual effect model, $e_{it} \sim i.i.d.N\left( {0,\sigma_{e}^{2} } \right),$ where *FR*_*i*_ and *GE*_*i*_ are dummy variables for France and Germany, respectively, AP1 = 1 if AP is “Mixed”, 0 otherwise; AP2 = 1 if AP is “Only Atypical”, 0 otherwise; the complement is when AP is “Only Typical”. *i* = 1,…,N is the individual dimension, and *t* = 1,…,5 is the time dimension. The indices *i* correspond to the patients, and *t* represents the visit number (1–5). *e*_*it*_ is an error term specific to individual *i* at visit *t*, and *u*_*i*_ is an error term specific to individual *i*. Here, it is assumed that corr (u_i_, *X*) = 0, which will be tested with the Hausman’s test.

To verify the strength of the estimates, the models were also estimated with the error terms *e*_*it*_ following an autoregressive process of order one, denoted AR [10].

The same models were also applied to the SF-6D utility measures using a similar methodology.

Finally, Ara et al. [4] recently showed that least-squares statistical models may perform well on the aggregate level, but that predicting preference-based utility values using partial proportional odds models (PPOM) and reconstructing the ED-5D global score from these sub-scores may perform better than using a conventional linear statistical model. In the case of schizophrenia data, the various probabilities are not affected by the same variables. Consequently, a more general and appropriate model, the universal multi-nomial logit model (UMLM) [31], may be used. The results of the UMLM may be useful to reconstruct the EQ-5D global score from the sub-scores and also to account for different tariffs depending on the countries. The model and the results are presented in online resources.

### Statistical analysis

Descriptive analyses were conducted for all variables, and correlation structures were examined.

In case of high correlation between explanatory variables, the relevant variables cannot be selected with respect to their statistical significance as it may result in the selection of collinear variables and estimation biases. Therefore, an alternative method was used for the variable selection: An approach similar to that of the principal components analysis (PCA) was conducted by selecting variables with the highest contribution to the variance. Contribution to the variance was assessed iteratively with removing the variable that had shown the highest contribution to the variance at the previous step.

To assess the selection bias, two sub-samples were considered: a sub-sample with no missing data and a sub-sample with missing values in at least one of the following variables: EQ-5D, PANSS_POS, PANSS_NEG, PANSS_PSY, age, sex, ATYP, CDSS, GAF, or BAS. No missing data for the country variable were found. Mean values for all the variables were compared between sub-samples, using Student tests or Chi-square tests, as appropriate.

To assess the predictive power of the developed models, two measures of error were calculated: the mean absolute error—the average of the absolute differences between the observed and the predicted values (MAE), and the root-mean-squared error—the square root of the average of the squared differences between the observed and predicted values (RMSE) [19].

In order to conduct a cross-validation, the sample was split randomly (each observation had a probability *p* to be selected in the training set, where *p* was chosen arbitrarily). Here, *p* = 0.5, *p* = 0.25 and *p* = 0.1 were chosen. However, the prediction error may vary depending on the sample partition. Consequently, to reduce the variability, multiple rounds of cross-validation were performed using different partitions, and the results were averaged among the rounds. A total of 1000 rounds were performed, and random selection was made for each round.

STATA^®^ software was used for all analyses.

## Results

Descriptive results are presented in Table 1. The mean patient age was 41.8 years. Males were more prevalent in the cohort (63 vs. 37 %). The mean PANSS was about 55, corresponding to “mildly ill” state.

**Table 1:** Descriptive statistics

Variable	Proxy for	Mean	SD	Min	Max
AGE (in years)	Age	41.80	10.88	18.70	66.62
Multi-attribute EuroQol EQ-5D score	Utility	0.76	0.26	−0.43	1.00
SF-6D score	Health-related QoL	0.71	0.13	0.30	1.00
Positive sub-score (PANSS_POS)	Symptoms severity	11.71	5.13	7	35
Negative sub-score (PANSS_NEG)	Symptoms severity	15.39	7.16	7	43
General psychopathology sub-score (PANSS_PSY)	Symptoms severity	27.87	9.65	16	71
Calgary Depression Scale for Schizophrenia (CDSS, range from 1 to 27)	Depressive symptoms	2.49	3.41	0	21
Global Assessment of Functioning (GAF, range 1–100)	Functioning	52.04	15.66	0	99
Barnes Akathisia Scale (BAS, range 1–100)	Extra-pyramidal symptoms	1.02	2.12	0	36

Variable	Proxy for	N	%		
Proportion of males	Gender	2593	63		
Proportion of French patients	Country	906	22		
Proportion of German patients		2099	51		
Proportion of UK patients		1111	27		
Proportion of mixed antipsychotic	Antipsychotic type	741	18		
Proportion of only atypical		1194	29		
Proportion of only typical		2181	53		

The correlation structure of the explanatory variables is summarized in Table SS1 (see Online resource). A strong correlation was observed between the PANSS_POS and PANSS_PSY scores (0.71), the PANSS_NEG and PANSS_PSY scores (0.69), and the PANSS_NEG and GAF scores (−0.5). The Hausman’s test showed that the correlation between the variables led to the co-linearity and would cause estimation biases, justifying the choice of the procedure for variable selection.

No significant difference was observed in the variables means between the sample without missing values and the sample with at least one missing value for all patients (see Table SS2 in Online resource). Both sub-samples were very close, thus confirming the absence of selection bias.

### Predictive model 1: using only the PANSS scores

The results for variable selection are presented in Table SS3 (see Online resource). The first relevant variable was PANSS_PSY with a contribution to *R*^2^ of 8.75 %. When PANSS_PSY effect had been removed, none of the variables demonstrated a large contribution to *R*^2^ (less or equal to 1 %). In the final model, sex and age variables were retained as well, because they were available in all the studies. PANSS_NEG had a negligible contribution to *R*^2^ (0.68 %) and was highly correlated with PANSS_PSY (69 %); it was therefore not used in the analyses.

The model estimates based on PANSS score only are presented in Table 2. PANSS_PSY and AGE variables had negative effects on the EQ-5D, whereas the male gender had a positive effect.

**Table 2:** EQ-5D predictive model estimates

EQ-5D	Coef.	Std. Err	z	*p* > |*z*|	[95 % Conf. interval]	
Model 1 (PANSS score only)						
Constant term	1.026055	0.0277925	36.92	0.000	0.971583	1.080527
PANSS_PSY	−0.0076876	0.0004386	−17.53	0.000	−0.0085473	−0.006828
Sex	0.0457537	0.0120427	3.80	0.000	0.0221504	0.0693569
Age	−0.0020646	0.0005313	−3.89	0.000	−0.0031059	−0.0010233
Model 2 (PANSS score and additional covariates)						
Constant term	1.011187	0.025353	39.88	0.000	0.9614961	1.060878
CDSS	−0.0192384	0.0011701	−16.44	0.000	−0.0215317	−0.016945
PANSS_PSY	−0.0047262	0.0004566	−10.35	0.000	−0.005621	−0.0038314
Age	−0.0023449	0.0004802	−4.88	0.000	−0.003286	−0.0014038
Sex	0.0322763	0.0108972	2.96	0.003	0.0109182	0.0536345

Model 1 (PANSS score only): number of observations = 4471, number of groups = 1181. *R* ^2^: within = 0.0394, between = 0.1500, overall = 0.1035. Wald test for significance (∼*χ* ^2^(3)) = 334.57 (*p* value = 0.0000). *σ*_*u*_ = 0.1697, *σ*_*e*_ = 0.1842, *ρ* = 0.4589 (fraction of variance due to *u*_*i*_). Breusch and Pagan Lagrangian multiplier test for random effects (∼*χ* ^2^(1)) = 1324.56 (*p* value = 0.0000)

Model 2 (PANSS score and additional covariates): number of observations = 4470, number of groups = 1181. *R* ^2^: within = 0.0615, between = 0.2958, overall = 0.1922. Wald test for significance (∼*χ* ^2^(3)) = 649.19 (*p* value = 0.0000). *σ*_*u*_ = 0.1458, *σ*_*e*_ = 0.1821, *ρ* = 0.3906 (fraction of variance due to *u*_*i*_). Breusch and Pagan Lagrangian multiplier test for random effects (∼*χ* ^2^(1)) = 979.44 (*p* value = 0.0000)

**Fig. 1:**
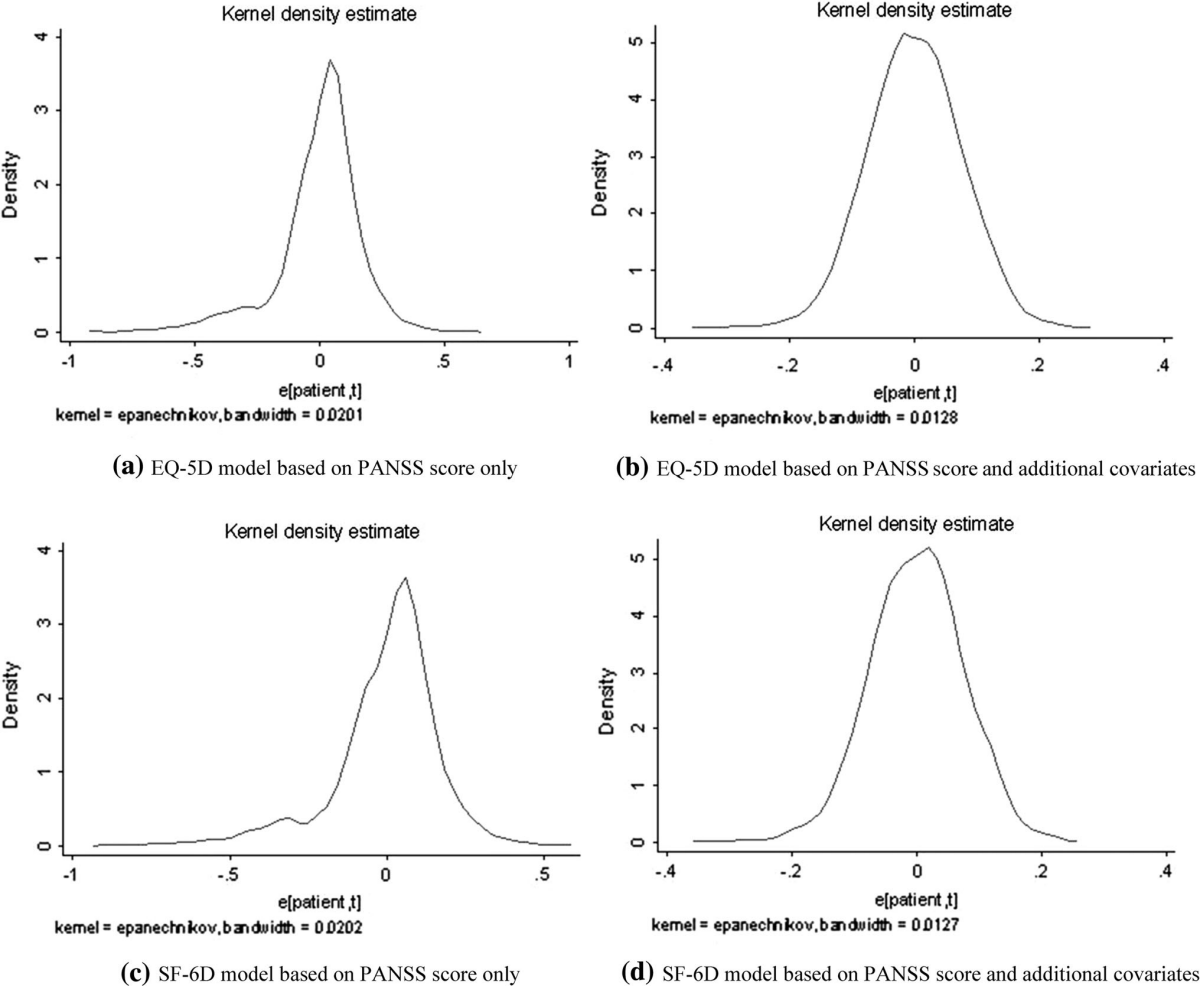
Probability density function estimates of the regressions, residuals. The figures present the residual probability density function estimates of the various regression models. The probability density functions are estimated using a nonparametric method based on a kernel density estimator

The model for the EQ-5D was also estimated with error terms following an AR [10] process. Small autocorrelation was observed (the estimated autocorrelation coefficient is 0.1786), but the estimated coefficients of the model with AR [10] error terms did not change with respect to the original model without AR [10] error terms. The Hausman’s test was applied and did not reveal any correlation between *u*_*i*_ and *X* (the *p* value is 0.1866). Consequently, the GLS estimator of the panel model was consistent. The normality hypothesis was rejected (the *p* values for skewness = 0, for kurtosis = 3, and for the Jarque–Bera test are 0.0000). However, the skewness and the kurtosis were not large (respectively −1.1125 and 5.8921), and the probability density function was roughly unimodal, as can be seen from the kernel density estimate of the residuals in Fig. 1a (Kernel density estimation is a nonparametric method for estimating the probability density function of a random variable.) Finally, the test of Ramsey reset for linearity of the model was applied, and the null hypothesis of linearity was rejected (the *p* value is 0.0130).

### Predictive model 2: using additional covariates

The results for variable selection are presented in Table SS3 (see Online resource). Three variables were considered relevant: CDSS (contribution to *R*^2^ = 16.27 %), PANSS_PSY (contribution to *R*^2^ = 3.10 %), and AGE (contribution to *R*^2^ = 1.30 %). In the final model, SEX (contribution to *R*^2^ = 0.45 %) variable was included as well.

The model estimates based on PANSS score and additional covariates are presented in Table 2. The Hausman’s test *p* value is 0.0000. Consequently, there existed a correlation between *u*_*i*_ and *X*, and the GLS estimator of the panel model was inconsistent. Fixed individual effect model estimates were then calculated and compared to the random individual effect model estimates (see Table SS4 in Online resource). The problem arose from CDSS variable, which might be correlated with the random individual effect u_i_ (the fixed effect estimate minus the random effect estimate = 0.00679, with standard error of 0.0007483). The fixed effect model was consistent under this hypothesis and led to a CDSS coefficient estimate of −0.0124, with a confidence interval of [−0.0151; −0.0097].

The model was also estimated with error terms following an AR [10] process. Small autocorrelation was observed (the estimated autocorrelation coefficient is 0.1706), but the estimated coefficients of the model did not change. The normality hypothesis was still rejected (the *p* values for testing skewness = 0, for testing kurtosis = 3, and for the Jarque–Bera test are 0.0000). However, the skewness and kurtosis were not substantial (−1.1350 and 5.8888, respectively), and the probability density function was roughly unimodal (see the kernel density estimate of the residuals in Fig. 1c). Finally, the Ramsey reset test for linearity was applied, and the null hypothesis of linearity was retained (the *p* value is 0.1645).

### Predictive models for the SF-6D

The same methodology used for the EQ-5D is used for the SF-6D.

Detailed results on the variable selection are available in Table SS2 (see Online resource).

The estimates for SF-6D model based on PANSS score only and on PANSS score and additional covariates are presented in Table 3. The results are similar to those for the EQ-5D. The PANSS_PSY negatively affects the utility measure, whereas male gender affects it positively.

**Table 3:** SF-6D predictive model estimates

SF-6D	Coef.	Std. Err	z	*p* > |*z*|	[95 % Conf. interval]	
Model 1 (PANSS score only)						
Constant term	0.7958439	0.0073481	108.31	0.000	0.781442	0.8102459
PANSS_PSY	−0.0037952	0.0002124	−17.86	0.000	−0.0042115	−0.0033788
Sex	0.0243601	0.0058699	4.15	0.000	0. 0128554	0.0358648
Model 2 (PANSS score and additional covariates)						
Constant term	0.8198619	0.0124882	65.65	0.000	0.7953856	0.8443382
CDSS	−0.009496	0.0005595	−16.97	0.000	−0.0105926	−0.0083993
PANSS_PSY	−0.0021323	0.0002253	−9.46	0.000	−0.002574	−0.0016907
BAS	−0.0040303	0.000773	−5.21	0.000	−0.0055454	−0.0025152
Age	−0.0008743	0.0002373	−3.68	0.000	−0.0013394	−0.0004092
Sex	0.0139095	0.0053836	2.58	0.003	0.0033579	0.0244612

Model 1 (PANSS score only): number of observations = 4338, number of groups = 1169. *R* ^2^: within = 0.0460, between = 0.1306, overall = 0.1036. Wald test for significance (∼*χ* ^2^(3)) = 327.86 (*p* value = 0.0000). *σ*_*u*_ = 0.0840, *σ*_*e*_ = 0.0856, *ρ* = 0.4904 (fraction of variance due to *u*_*i*_). Breusch and Pagan Lagrangian multiplier test for random effects (∼*χ* ^2^(1)) = 1653.046 (*p* value = 0.0000)

Model 2 (PANSS score and additional covariates): number of observations = 4258, number of groups = 1166. *R* ^2^: within = 0.0812, between = 0.2908, overall = 0.2166. Wald test for significance (∼*χ* ^2^(3)) = 704.73(*p* value = 0.0000). *σ*_*u*_ = 0.0687, *σ*_*e*_ = 0.0874, *ρ* = 0.3819 (fraction of variance due to *u*_*i*_). Breusch and Pagan Lagrangian multiplier test for random effects (∼*χ* ^2^(1)) = 1212.22(*p* value = 0.0000)

In case of SF-6D model based on PANSS score and additional covariates, a better estimate for CDSS was provided by the fixed effect model: −0.0067173 with a confidence interval of [−0.0080064, −0.0054282]. Again, the results are similar to those of EQ-5D, suggesting that both the algorithms are robust.

The same specification tests as for the EQ-5D were applied to the both SF-6D models. Minor autocorrelation was observed; however, the estimated coefficients of the model did not change. The normality hypothesis was again rejected (the *p* value for testing skewness = 0 is 0.004, the *p* value for testing kurtosis = 3 is 0.0000, and the *p* value for the Jarque–Bera test is 0.0000). However, the skewness and the kurtosis were close to 0 and 3, respectively. In addition, the probability density function was unimodal and close to the Gaussian probability density function (see the kernel density estimate of the residuals in Fig. 1b, d). The null hypothesis of linearity was rejected (Ramsey reset *p* value = 0.0000).

### Measuring predictive ability and cross-validation

The predictive errors for the various models are presented in Table 4. The results for the cross-validation are reported in the same table for facilitating the comparison. Various proportions *p* of the observations used to construct the training set were chosen to verify the strength of the models; *p* = 0.5, 0.25, and 0.1 were tested.

**Table 4:** Predictive error results: Predicted versus observed

Dependent variable	Independent variables	Mean	Min	Max	Corr	RMSE	MAE
EQ-5D	Observed	0.758	−0.429	1	1	0	0
EQ-5D	Only PANSS	0.752	0.369	0.907	0.321	0.246	0.182
EQ-5D	Only PANSS^a^	0.753	0.362	0.905	0.328	0.250	0.184
EQ-5D	Only PANSS^b^	0.755	0.367	0.924	0.316	0.248	0.182
EQ-5D	Only PANSS^c^	0.735	0.219	0.930	0.317	0.246	0.186
EQ-5D	Additional	0.753	0.251	0.920	0.438	0.234	0.173
EQ-5D	Additional^a^	0.754	0.314	0.917	0.442	0.234	0.173
EQ-5D	Additional^b^	0.753	0.230	0.919	0.439	0.235	0.174
EQ-5D	Additional^c^	0.750	0.114	0.910	0.433	0.234	0.173
SF-6D	Observed	0.707	0.301	1	1	0	0
SF-6D	Only PANSS	0.704	0.514	0.775	0.328	0.119	0.098
SF-6D	Only PANSS^a^	0.706	0.552	0.774	0.342	0.119	0.098
SF-6D	Only PANSS^b^	0.702	0.552	0.773	0.342	0.119	0.097
SF-6D	Only PANSS^c^	0.703	0.512	0.765	0.325	0.120	0.098
SF-6D	Additional	0.704	0.466	0.782	0.465	0.112	0.091
SF-6D	Additional^a^	0.708	0.441	0.788	0.454	0.112	0.092
SF-6D	Additional^b^	0.703	0.422	0.785	0.457	0.112	0.091
SF-6D	Additional^c^	0.706	0.399	0.786	0.466	0.112	0.092

Cross-validation

^a^Training set: 50 % of the original sample (drawn randomly), validation set: 50 %

^b^Training set: 25 % of the original sample (drawn randomly), validation set: 75 %

^c^Training set: 10 % of the original sample (drawn randomly), validation set: 90 %

RMSE and the MAE values were similar for all analyses, showing no increase depending on the training set size. It can be concluded that the models are robust and can be used to predict the utility measure for other datasets.

## Discussion

This study aimed to build a model to map the demographic and clinical measures of patients with schizophrenia to EQ-5D index. Although the EQ-5D is currently recommended by NICE [32] for use in economic evaluation, it remains largely under-utilized in clinical trials for schizophrenia. As suggested by the NICE [32], mapping can be used when EQ-5D is not included in clinical trials. The proposed mapping functions can constitute the first step in promoting the assessment of utility values in schizophrenia as required in cost-utility analyses. Our findings suggest that the mapping relationship between the socio-demographic, clinical characteristics, and EQ-5D is reliable and robust.

From a clinical perspective, age, gender, PANSS psychopathology score, and CDSS score are the most important predictors associated with EQ-5D. These findings are consistent with those of the studies focusing on non-preference-based health status measures. Age negatively affects the utility measure. This is in line with Kemmler et al. [25] results, showing that social problems, isolation, and stigmatization of patients with schizophrenia tend to increase with age. Male gender positively affects utility measures. This finding appears to be consistent with the general literature, in which the quality of life of female patients is often reported to be lower than that of men, especially with regard to psychological and mental health domains [21, 35]. Regarding the influence of the PANSS scores, PANSS psychopathology factor was found to negatively affect the utility (*p* < 0.001), whereas the PANSS positive and negative factors do not affect utility measures. Similarly to the PANSS psychopathology factor, CDSS score negatively affects the utility (*p* < 0.001). These findings concur with those of several studies and meta-analyses that reveal that symptoms have only a modest relationship with quality of life and that general psychopathology symptoms (e.g. anxiety and depression) were the most important predictors [8, 16, 20]. Finally, extra-pyramidal symptoms (BAS) are associated with lower utility measures. In point of fact, the burden of the side effects has been extensively explored as a predictor of poor medication adherence, relapse, and poor QoL [3, 18, 21].

From a methodological aspect, the high co-linearity among the explanatory variables led to the inappropriateness of variable selection based on the statistical significance. A specific procedure employing the principle of PCA was then developed and applied. Inconsistent parameter estimates were obtained for the EQ-5D random effect model based on PANSS score and additional covariates because of the correlation between some explanatory variables and the errors terms. The estimates were then recalculated using the fixed effects panel model, which provided consistent results. Additionally, a number of other specification tests were performed in order to provide the evidence that the proposed mappings model is well specified and reliable. Finally, the proposed approach was also tested with SF-6D. The results were coherent with those obtained for EQ-5D confirming the robustness of the method. In their paper, McCrone et al. [30] showed that from an analytical perspective, the SF-6D has advantages over the EQ-5D due to its normal distribution and the lack of ceiling effect. However, both measures produced similar mean utility scores, and further comparisons of the EQ-5D and SF-6D were required. Additionally, the prediction errors were calculated using RMSE and MAE. Their values remained moderate. For the EQ-5D model, RMSE was around 0.25 and MAE was approximately 0.18. SF-6D model prediction errors were quite lower (RMSE and MAE were about 0.12 and 0.10, respectively). The cross-validation results confirmed the stability of the models. It may be concluded that the models are applicable to predict utility measures.

It should be noted that the EQ-5D value theoretically range from minus infinite to one. In the original data, the range is [−0.429; 1.000]; however, the range of predicted values is [0.114; 0.930]. A similar pattern is observed for SF-6D. Predictive models generate predictions of the conditional mean, but not of the conditional variance and thus may compress the range of individual predicted values. The variance can be accounted for by using the predicting interval instead of the predicting value. Siani et al. [37] show how to account for uncertainty in the context of mapping prediction.

Regarding the analysis limitations, the representativeness of the sample should first be discussed. Although the sampling procedure for the EuroSC aimed to provide a representative patients sample, this cohort included mostly paranoid schizophrenia and is characterized by long-term illness. Moreover, the difference in severity between excluded and included patients was observed (with higher clinical severity in excluded patients). Further analysis is therefore required, using larger and more diverse groups of patients. However, the large sample size of the presented study and the longer follow-up allowed overcoming the limitations of past studies [26].

## Conclusion

Because treatments for schizophrenia have significant effects on the quality of life of patients, reliable methods for economic evaluations are needed to account for effects of treatment, to assess utility values, and to calculate QALYs for further cost-utility analyses. This paper proposes reliable, accurate, and easy-to-apply mapping models for EQ-5D index based on demographic and clinical measures in schizophrenia. An advantage of these mapping functions is the use of generally available in clinical trials data, such as PANSS score, which expands its applicability.

## Electronic supplementary material

Below is the link to the electronic supplementary material.

Supplementary material 1 (DOCX 19 kb)

Supplementary material 2 (DOCX 18 kb)

Supplementary material 3 (DOCX 20 kb)

Supplementary material 4 (DOCX 17 kb)

Supplementary material 5 (DOCX 46 kb)
