# Supplementary material for: Predictive models to estimate utility from clinical questionnaires in schizophrenia: findings from EuroSC
Source: Qual Life Res. 2015 Sep 18;25:925–34. doi: 10.1007/s11136-015-1120-6 (PMC4830865; doi:10.1007/s11136-015-1120-6)
Supplement: Supplementary file 1 — Supplementary material 1 (DOCX 19 kb) [file 11136_2015_1120_MOESM1_ESM.docx]

**Online resource 1. Correlation structure**

|  | EQ-5D | PANS_POS | PANS_NEG | PANS_PSY | AGE | SEX | FR | GE | Mix | Only Typiq | Only Atypiq | CDSS | GAF | BAS | COMP |
| --- | --- | --- | --- | --- | --- | --- | --- | --- | --- | --- | --- | --- | --- | --- | --- |
| EQ-5D | 1.00 |  |  |  |  |  |  |  |  |  |  |  |  |  |  |
| PANS_POS | -0.22 | 1.00 |  |  |  |  |  |  |  |  |  |  |  |  |  |
| PANS_NEG | -0.11 | 0.42 | 1.00 |  |  |  |  |  |  |  |  |  |  |  |  |
| PANS_PSY | -0.30 | 0.71 | 0.69 | 1.00 |  |  |  |  |  |  |  |  |  |  |  |
| AGE | -0.09 | 0.00 | 0.02 | 0.00 | 1.00 |  |  |  |  |  |  |  |  |  |  |
| SEX | 0.08 | 0.08 | 0.11 | 0.06 | -0.15 | 1.00 |  |  |  |  |  |  |  |  |  |
| FR | -0.02 | 0.24 | 0.28 | 0.36 | -0.08 | 0.07 | 1.00 |  |  |  |  |  |  |  |  |
| GE | -0.01 | -0.14 | 0.02 | -0.11 | 0.06 | -0.10 | -0.53 | 1.00 |  |  |  |  |  |  |  |
| Mix | -0.08 | 0.12 | 0.10 | 0.13 | -0.07 | 0.05 | 0.02 | -0.02 | 1.00 |  |  |  |  |  |  |
| Only Typiq | -0.01 | -0.05 | -0.02 | -0.03 | 0.27 | -0.02 | 0.00 | 0.04 | -0.50 | 1.00 |  |  |  |  |  |
| Only Atypiq | 0.08 | -0.04 | -0.07 | -0.08 | -0.24 | -0.02 | -0.02 | -0.02 | -0.30 | -0.68 | 1.00 |  |  |  |  |
| CDSS | -0.41 | 0.22 | 0.20 | 0.43 | -0.04 | -0.07 | 0.05 | 0.01 | 0.10 | -0.05 | -0.03 | 1.00 |  |  |  |
| GAF | 0.22 | -0.40 | -0.50 | -0.45 | -0.14 | -0.07 | 0.08 | -0.20 | -0.15 | 0.02 | 0.11 | -0.24 | 1.00 |  |  |
| BAS | -0.13 | 0.15 | 0.09 | 0.16 | 0.06 | 0.05 | -0.07 | 0.01 | 0.09 | 0.02 | -0.10 | 0.17 | -0.18 | 1.00 |  |
| COMP | -0.01 | -0.02 | -0.02 | 0.02 | -0.12 | -0.02 | 0.25 | -0.38 | 0.01 | -0.03 | 0.02 | 0.02 | 0.18 | -0.03 | 1.00 |
