# Supplementary material for: Predictive models to estimate utility from clinical questionnaires in schizophrenia: findings from EuroSC
Source: Qual Life Res. 2015 Sep 18;25:925–34. doi: 10.1007/s11136-015-1120-6 (PMC4830865; doi:10.1007/s11136-015-1120-6)
Supplement: Supplementary file 2 — Supplementary material 2 (DOCX 18 kb) [file 11136_2015_1120_MOESM2_ESM.docx]

**Online resource 2. Comparison between the subsample without missing data and the subsample with at least one missing value for all patients**

| Subsample | No missing value | Missing values |
| --- | --- | --- |
| Age mean (SD) | 41.80 (0.17) | 42.29 (0.43) |
| EQ-5D mean (SD) | 0.76 (0.0040) | 0.75 (0.014) |
| SF-6D mean (SD) | 0.71 (0.0020) | 0.71 (0.0061) |
| PANSS_POS mean (SD) | 11.71 (0.080) | 12.76 (0.25) |
| PANSS_NEG mean (SD) | 15.39 (0.11) | 15.42 (0.29) |
| PANSS_PSY mean (SD) | 27.81 (0.15) | 28.68 (0.40) |
| CDSS mean (SD) | 2.49 (0.053) | 2.22 (0.12) |
| GAF mean (SD) | 52.04 (0.053) | 51.48 (0.67) |
| BAS mean (SD) | 1.02 (0.033) | 0.77 (0.096) |
| Sex N (%) | 2 593 (63%) | 425 (57%) |
| Atyp “Mix” N (%) | 741 (18%) | 127 (17%) |
| Atyp “Only Typiq” N (%) | 2 181 (53%) | 410 (55%) |
| Atyp “Only Atypiq” N (%) | 1 194 (29%) | 216 (29%) |
