# Supplementary material for: Predictive models to estimate utility from clinical questionnaires in schizophrenia: findings from EuroSC
Source: Qual Life Res. 2015 Sep 18;25:925–34. doi: 10.1007/s11136-015-1120-6 (PMC4830865; doi:10.1007/s11136-015-1120-6)
Supplement: Supplementary file 3 — Supplementary material 3 (DOCX 20 kb) [file 11136_2015_1120_MOESM3_ESM.docx]

**Online resource 3. Variable selection**

| EQ-5D | | EQ-5D-PANSS PSY | | EQ-5D-PANSS_PSY-SEX | | EQ-5D-PANSS_PSY-SEX-AGE | |
| --- | --- | --- | --- | --- | --- | --- | --- |
| Variable | $R^{2}$ | Variable | $R^{2}$ | Variable | $R^{2}$ | Variable | $R^{2}$ |
|  | | | | | | | |
| **Model 1 (PANSS score only)** | | | | | | | |
| PANSS_PSY | 0.0875 | SEX | 0.0116 | AGE | 0.0072 | PANSS_NEG | 0.0068 |
| PANSS_POS | 0.0429 | AGE | 0.0102 | AGE2 | 0.0069 | FR | 0.0045 |
| PANSS_NEG | 0.0134 | AGE2 | 0.0098 | PANSS_NEG | 0.0055 | GE | 0.0009 |
| AGE | 0.0095 | PANSS_NEG | 0.0069 | FR | 0.0050 | PANSS_POS | 0.0001 |
| AGE2 | 0.0084 | FR | 0.0059 | GE | 0.0010 | AGE2 | 0 |
| SEX | 0.0071 | GE | 0.0017 | PANSS_POS | 0.0002 |  |  |
| FR | 0.0007 | PANSS_POS | 0.0000 |  |  |  |  |
| GE | 0.0001 |  |  |  |  |  |  |
|  | | | | | | | |
| **Model 2 (PANSS score and additional covariates)** | | | | | | | |
| CDSS | 0.1627 | PANSS_PSY | 0.0310 | AGE | 0.0130 | GAF | 0.0056 |
| PANSS_PSY | 0.0875 | GAF | 0.0276 | AGE2 | 0.0130 | PANSS_NEG | 0.0048 |
| GAF | 0.0522 | PANSS_POS | 0.0240 | GAF | 0.0092 | SEX | 0.0045 |
| PANSS_POS | 0.0429 | AGE | 0.0134 | SEX | 0.0079 | BAS | 0.0035 |
| BAS | 0.0164 | AGE2 | 0.0128 | BAS | 0.0040 | FR | 0.0024 |
| PANSS_NEG | 0.0134 | BAS | 0.0070 | ATYP2 | 0.0038 | ATYP2 | 0.0012 |
| AGE | 0.0095 | ATYP2 | 0.0051 | PANSS_NEG | 0.0038 | ATYP1 | 0.0011 |
| AGE2 | 0.0084 | SEX | 0.0050 | FR | 0.0031 | PANSS_POS | 0.0010 |
| SEX | 0.0071 | PANSS_NEG | 0.0031 | PANSS_POS | 0.0012 | GE | 0.0005 |
| ATYP2 | 0.0053 | ATYP1 | 0.0019 | GE | 0.0008 | AGE2 | 0.0000 |
| ATYP1 | 0.0050 | FR | 0.0001 | ATYP1 | 0.0007 |  |  |
| FR | 0.0007 | GE | 0.0001 |  |  |  |  |
| GE | 0.0001 |  |  |  |  |  |  |
