# Supplementary material for: Predictive models to estimate utility from clinical questionnaires in schizophrenia: findings from EuroSC
Source: Qual Life Res. 2015 Sep 18;25:925–34. doi: 10.1007/s11136-015-1120-6 (PMC4830865; doi:10.1007/s11136-015-1120-6)
Supplement: Supplementary file 4 — Supplementary material 4 (DOCX 17 kb) [file 11136_2015_1120_MOESM4_ESM.docx]

**Online resource 4. Hausman’s test: Difference between fixed and random effect model estimates**

| Variable | Fixed effect model | Random effect model | Fixed – Random effects | |
| --- | --- | --- | --- | --- |
| CDSS | -0.0124484 | -0.0192384 | 0.00679 | 0.0007483 |
| PANSS_PSY | -0.0048544 | -0.0047262 | -0.0001282 | 0.0004532 |
| AGE | 0.0019578 | -0.0023449 | 0.0043027 | 0.0040731 |

Fixed effect: consistent under Ho and Ha.

Random effect: inconsistent under Ha, consistent under Ho.
