# Supplementary material for: Predictive models to estimate utility from clinical questionnaires in schizophrenia: findings from EuroSC
Source: Qual Life Res. 2015 Sep 18;25:925–34. doi: 10.1007/s11136-015-1120-6 (PMC4830865; doi:10.1007/s11136-015-1120-6)
Supplement: Supplementary file 5 — Supplementary material 5 (DOCX 46 kb) [file 11136_2015_1120_MOESM5_ESM.docx]

**Predictive model 3: Predicting sub-scores using only the PANSS scores**

We also derived a model for predicting sub-scores using only the PANSS scores:


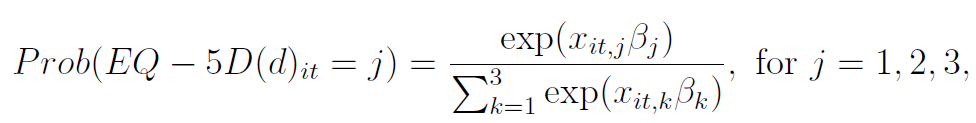


where EQ-5D(d) is the domain number d = 1,…, 5 of the EQ-5D, j is the domain level, and xit,j is the set of variables PANSS_POSit, PANSS_NEGit, PANSS_PSYit, AGEit, AGE2it, SEXi, FRi, GEi or a subset of these variables depending on j. This model is similar to the universal multinomial logit model (McFadden, 1973) but extended to the panel framework.

In practice, using Stata software, the predictive Model 3 is estimated by combining two classical panel logit models (Bertschek and Lechner, 1998) to get three levels of answer. A panel logit model with random individual effects is first applied for estimating the probability that EQ-5D(d)it = 1 against the probability that EQ-5D(d)it = 2 or 3 (which is a classical panel dichotomous model). When EQ-5D(d)it $\neq$ 1, another panel logit model with random individual effects is applied to estimate the probability that EQ-5D(d)it = 2 against the probability that EQ-5D(d)it = 3. The combination of the two dichotomous models leads to a polytomous model with flexible explanatory variables (as in the conditional logit model, see Chapter 3 in Maddala, 1983) and parameters depending on EQ-5D(d) level j (as in the universal multinomial logit model).

The coefficient estimates are summarised in Table SS5. The complete results are available by request to the corresponding author.

**Table SS5. Predictive model 3: explaining sub-scores of the EQ-5D using only the PANSS scores**

| Domain | EQ-5D(1) | | EQ-5D(2) | | EQ-5D(3) | | EQ-5D(4) | | EQ-5D(5) | |
| --- | --- | --- | --- | --- | --- | --- | --- | --- | --- | --- |
|  | mobility | | self-care | | usual activities | | pain/discomfort | | anxiety/depression | |
| Level | 2\|-1 | 1 | 2\|-1 | 1 | 2\|-1 | 1 | 2\|-1 | 1 | 2\|-1 | 1 |
| Constant term |  | 5.2828 | 6.0761 | 5.9866 | 4.9867 | 3.2671 | 6.9469 | 3.4402 | 4.9348 | 2.3770 |
| PANSS-POS |  |  |  |  |  |  |  |  |  |  |
| PANSS-NEG |  |  | -0.1108 |  | -0.0952 |  |  |  |  |  |
| PANSS-PSY |  | -0.0432 |  | -0.0790 |  | -0.0767 | -0.0550 | -0.0397 | -0758 | -0.0792 |
| AGE |  | -0.0473 |  | -0.0232 |  | 0.0096 | -0.0397 | -0.0409 |  |  |
| SEX |  | 0.5643 |  |  |  |  |  |  |  |  |
| FR |  |  |  | 1.3462 |  | 1.3888 |  |  |  |  |
| GE |  |  | 1.0144 | -0.8791 | 0.8440 |  |  |  |  |  |
| Sample size | 836 | 4524 | 891 | 4545 | 1577 | 4536 | 1814 | 4543 | 2158 | 4591 |

This table presents the parameter estimates $\hat{\beta_{j}}$ of the model described above.

Column “1” corresponds to the dichotomous logit model explaining P(Domainit = 1) against P(Domainit $\neq$ 1), where 1 is the best level of the domain. When the level is not 1, Column 2|-1 corresponds to the dichotomous logit model explaining P(Domainit = 2 | Domainit $\neq$ 1) against P(Domainit = 3 | Domainit $\neq$ 1), where 3 is the worst level of the domain.

For the EQ-5D(1) domain (mobility), the PANSS PSY and AGE negatively affect the probability of State 1 to occur (the best one), whereas male gender affects it positively. No conclusion can be reached regarding the variables that affect the probability of States 2 and 3 to occur because there are only three observations in State 3, which is not enough to estimate the parameters. For the EQ-5D(2) domain (self-care), the PANSS PSY and AGE negatively affects the probability that State 1 occurs. However, the PANSS NEG determines if the health state is 3 (the worst state). There is some specificity with respect to the countries. The results are similar for the EQ-5D(3) domain (usual activities). For the EQ-5D(4) domain (pain/discomfort) and the EQ-5D(5) domain (anxiety/depression), the PANSS PSY has a negative effect on all the health states. A larger PANSS PSY is correlated with a worse health state (close to 3). For the EQ-5D(4) domain, the AGE has also a negative effect.
